# Var2CSA Minimal CSA Binding Region Is Located within the N-Terminal Region

**DOI:** 10.1371/journal.pone.0020270

**Published:** 2011-05-19

**Authors:** Anand Srivastava, Stéphane Gangnard, Sébastien Dechavanne, Farroudja Amirat, Anita Lewit Bentley, Graham A. Bentley, Benoît Gamain

**Affiliations:** 1 Institut Pasteur, Unité d'Immunologie Structurale, F-75015 Paris, France; 2 CNRS, URA2185, F-75015 Paris, France; 3 INSERM, U665, F-75739 Paris, France; 4 Institut National de la Transfusion Sanguine, F-75739 Paris, France; 5 Univ Paris Diderot, Sorbonne Paris Cité, Protéines de la membrane érythrocytaire, UMR-S665, F-75739 Paris, France; Univ. Georgia, United States of America

## Abstract

Var2CSA, a key molecule linked with pregnancy-associated malaria (PAM), causes sequestration of *Plasmodium falciparum* infected erythrocytes (PEs) in the placenta by adhesion to chondroitin sulfate A (CSA). Var2CSA possesses a 300 kDa extracellular region composed of six Duffy-binding like (DBL) domains and a cysteine-rich interdomain region (CIDRpam) module. Although initial studies implicated several individual var2CSA DBL domains as important for adhesion of PEs to CSA, new studies revealed that these individual domains lack both the affinity and specificity displayed by the full-length extracellular region. Indeed, recent evidence suggests the presence of a single CSA-binding site formed by a higher-order domain organization rather than several independent binding sites located on the different domains. Here, we search for the minimal binding region within var2CSA that maintains high affinity and specificity for CSA binding, a characteristic feature of the full-length extracellular region. Accordingly, truncated recombinant var2CSA proteins comprising different domain combinations were expressed and their binding characteristics assessed against different sulfated glycosaminoglycans (GAGs). Our results indicate that the smallest region within var2CSA with similar binding properties to those of the full-length var2CSA is DBL1X-3X. We also demonstrate that inhibitory antibodies raised in rabbit against the full-length DBL1X-6ε target principally DBL3X and, to a lesser extent, DBL5ε. Taken together, our results indicate that efforts should focus on the DBL1X-3X region for developing vaccine and therapeutic strategies aimed at combating PAM.

## Introduction

Pregnancy-associated malaria (PAM) causes adverse pregnancy outcomes, including anemia and hypertension in first-time pregnant women, and low birth weight due to premature delivery and fetal growth restriction, which are associated with a higher risk of fetal and neonate morbidity and mortality [1], [2]. Complications arising from PAM have been attributed to massive accumulation of *Plasmodium falciparum*-infected erythrocytes (PEs) in the placenta mediated by adhesion to chondroitin sulfate A (CSA) [3]. Significantly, after one or two pregnancies, women acquire transcendent antibodies recognizing placental PEs from different geographic regions that inhibit placental adhesion, thus correlating with protection against malaria [4], [5].

Several lines of evidence support var2CSA, a member of the *Plasmodium falciparum* Erythrocyte Membrane Protein 1 (PfEMP1) adhesins encoded by the *var* gene family [6], [7], [8], as the leading PAM vaccine candidate. Indeed, var2CSA is the only *var* gene transcribed in CSA-binding laboratory isolates and placental PEs [9], [10], [11], [12], [13], [14]. Importantly, gene disruption studies have clearly demonstrated that var2CSA is the primary *var* gene responsible for the CSA-binding phenotype, as Δvar2CSA mutant clones either did not recover the CSA-binding phenotype [15] or otherwise switched to low affinity CSA binders that no longer reacted in a gender-specific manner with multigravid sera [16]. These mutant parasites were unable to express any other ligand that promoted extensive sequestration in placental tissue [16], [17]. Finally, antibodies to var2CSA-expressing isolates and to var2CSA recombinant proteins have been consistently associated with protection against malaria during pregnancy [11], [18], [19], [20]. World-wide parasite isolates analysed so far contain at least one var2CSA ortholog with an amino acid identity ranging from 54% to 94% and distinct, as well as conserved, epitopes [13], [21], [22], [23].

**Figure 1:**
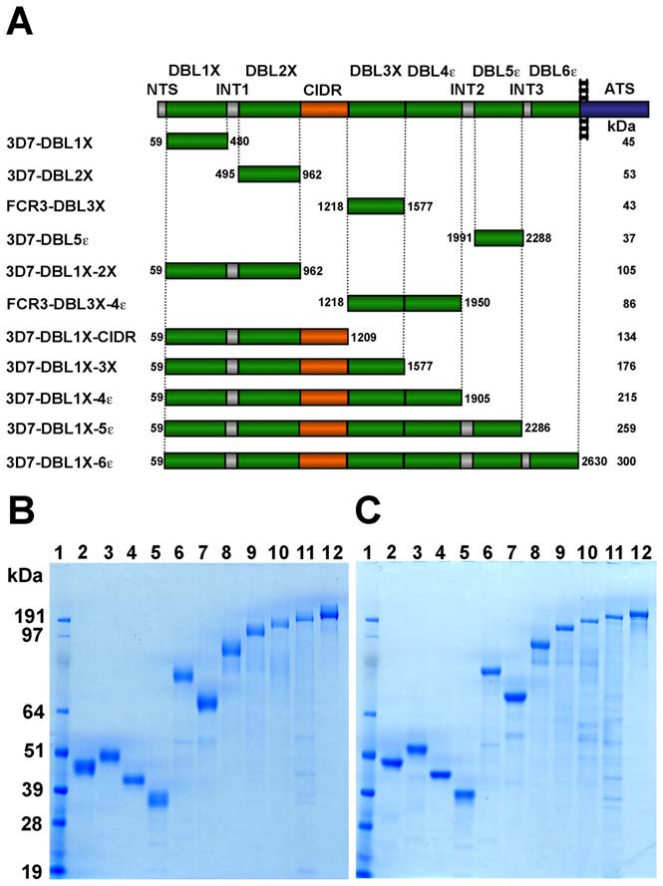
Various var2CSA recombinant proteins expressed in HEK293 cells and *E. coli*. (A). Schematic view of the var2CSA domain organization and sequence limits of the recombinant domains studied (3D7-DBL1X, 3D7-DBL2X, FCR3-DBL3X, 3D7-DBL5ε, 3D7-DBL1X-2X, FCR3-DBL3X-4ε, 3D7-DBL1X-CIDR, 3D7-DBL1X-3X, 3D7-DBL1X-4ε, 3D7-DBL1X-5ε and 3D7-DBL1X-6ε). Var2CSA comprises six DBL domains (DBL1X to DBL6ε), a CIDRpam domain and three inter-domain regions (INT1-3) in the extracellular region, together with a trans-membrane segment and acidic C terminus sequence (ATS). DBL1X, DBL2X, DBL3X, DBL4ε, DBL5ε and DBL6ε are shown in green; CIDR in orange; N-terminal sequence (NTS) and inter-domain regions (INT) in grey; the trans-membrane and ATS regions in blue. The length of each bar corresponds to the domain size. (B). Purification of var2CSA derived proteins expressed in HEK293 and *E. coli*. Nu-SDS-PAGE Precast 4–12% Bis-Tris gel under nonreducing (B) and reducing (C) conditions was loaded with purified recombinant proteins. Lane 1: Marker, lane 2: 3D7-DBL1X, lane 3: 3D7-DBL2X, lane 4: FCR3-DBL3X, lane 5: 3D7-DBL5ε, lane 6: 3D7-DBL1X-2X, lane 7: FCR3-DBL3X-4ε, lane 8: 3D7-DBL1X-CIDR, lane 9: 3D7-DBL1X-3X, lane 10: 3D7-DBL1X-4ε, lane 11: 3D7-DBL1X-5ε and lane 12: 3D7-DBL1X-6ε. Proteins were visualized with Coomassie blue.

Var2CSA is a 350 kDa transmembrane protein with a 300 kDa extracellular region composed of six Duffy-binding-like (DBL) domains and a cysteine-rich interdomain region (CIDRpam) module, as well as short interdomain regions (Fig. 1A) [24], [25]. From *in vitro* binding assays, the CSA-binding properties have been mapped to several var2CSA domains, namely DBL2X, DBL3X, DBL5ε, and DBL6ε [14], [26], [27]. These studies suggested that the various CSA-binding domains might function independently of each other by forming multivalent interactions that together cause placental sequestration by avidity effects. However, more recent studies revealed that these individual domains have low affinity to CSA and that they can also bind to other sulfated glycosaminoglycans (GAGs), in some cases with higher affinity than to CSA [28]. Moreover, while most of these individual domains elicited antibodies that reacted with CSA-binding parasite isolates, only few induced an adhesion-blocking response [29], [30], [31], [32], [33], suggesting that individual domains are not sufficient to exhibit the full binding phenotype.

Recently, we and others have shown that the complete extracellular region of PfEMP1 possesses a higher-order organisation that is determined by well defined inter-domain contacts and is a prerequisite for creating the native high affinity CSA-specific binding site [34], [35]. Indeed, unlike the individual DBL domains, the full-length var2CSA extracellular region binds with high affinity and specificity to CSA. In addition, small angle X-ray scattering and analytical ultracentrifugation experiments showed that var2CSA forms a more compact structure than expected from an extended “beads-on-a-string” model [34], favoring the view that a single high affinity CSA-binding site is formed by the association of several var2CSA domains rather than an ensemble of independent sites residing on individual domains.

Although the full-length protein possesses binding properties characteristic of placental PEs, its size and polymorphism pose a challenge for vaccine development. There is therefore a need to identify which domain or combination of domains is required to form the high affinity CSA-binding site. Such knowledge would provide a rational basis for accelerating vaccine and therapeutic developments aimed at blocking the adhesion of CSA-binding parasites to the placenta. In order to address this question, we expressed various var2CSA DBL domains and multi-domain proteins in HEK293 and *E. coli* heterologous expression systems and tested their binding properties to CSA and other sulfated GAGs. In addition, we used these recombinant proteins to map the domains recognized by the inhibitory IgG raised in rabbits against the full-length extracellular region of var2CSA using antibody depletion and elution experiments.

Our results suggest that the high affinity CSA-binding site lies within the DBL1X-3X segment of var2CSA and that DBL3X and, to some extent, DBL5ε are the principal targets of the inhibitory antibodies. Taken together, our results indicate that DBL3X is an important target for inhibitory antibodies and that strategies aimed at blocking PE adhesion to CSA should focus on the N-terminal region of var2CSA. These results present an important new step towards the design of vaccine and therapeutic strategies to combat PAM.

## Methods

### Ethics statement

All animal work was conducted according to relevant national and international guidelines. Immunizations were performed by a custom vendor (Proteogenix, France), and all animal experiments were approved and conducted in accordance with the Institut Pasteur and Proteogenix Biosafety Committees. Animals were housed under controlled laboratory conditions by qualified personnel who were licensed by the French Agricultural Ministry (agreement B 75 15-08 dated May 22, 2008). All researchers performing animal experiments in this study were directly responsible for the experimental protocols and had obtained individual licenses from the French Ministry of Agriculture.

### Expression and Purification of Recombinant Protein

#### (i) HEK 293-F cell Recombinant Protein Expression and Purification

Synthetic *var2csa* genes for 3D7-DBL1X-3X (residues 59–1577) and 3D7-DBL4ε-6ε (residues 1578–2630) (accession PFL0030c) were designed with optimized codons for human cell expression, as previously described [34], and were cloned into the pTT3 vector [36]. pTT3-3D7-DBL1X-6ε (residues 59–2630) were prepared by incorporation of 3D7-DBL4ε-6ε into pTT3-3D7-DBL1X-3X using compatible restriction sites. Genes encoding 3D7-DBL1X (residues 59–430) and 3D7-DBL1X-CIDR (residues 59–1209) were PCR amplified from pTT3-3D7-DBL1X-3X and cloned into the pTT3 vector using EcoRI/HindIII restriction sites for expression in FreeStyle 293-F cells, as previously described (Invitrogen) [34]. The oligonucleotide primers used for amplification were as follows (restriction sites are represented in lower case): 3D7-DBL1X: DBL1XF (5′-AGC gaattc ATG GAG ACC GAC ACC C-3′) and 3D7-DBL1XR (5′-CAC aagctt CCC GCT TTC ATT GCA GTT GCA CAC-3′); 3D7-DBL1X-CIDR: 3D7-DBL1X-CIDRF (5′-AGC gaattc ATG GAG ACC GAC ACC C-3′) and 3D7-DBL1X-CIDRR (5′-CTC aagctt GCT GGT CTC AGA GCT TTT CAT C -3′). Genes encoding 3D7-DBL1X-4ε (residues 59–1905) and 3D7-DBL1X-5ε (residues 59–2286) were obtained by PCR amplification of 3D7-DBL4ε and 3D7-DBL4ε-5ε from pTT3-3D7-DBL1X-6ε prior to cloning into pTT3-DBL1X-3X, using HindIII/XbaI restrictions sites. The oligonucleotide primers used to amplify were as follows (restriction sites are represented in lower case): DBL4εF (5′- AAC aagctt CTG TGC CAC GAG AAA GG -3′) and DBL4εR (5′-GTT tctaga GTT CTT GCA GCT ACA GCA GAT G-3′), and DBL5εR (5′- CTT tctaga GTC ATT GAA GCC GCA GGG GCA C-3′) primers, respectively.

The pTT3 vector contains an N-terminal murine Ig κ-chain leader sequence and a C-terminal hexa-His tag. FreeStyle 293-F cells (Invitrogen) were grown in Freestyle 293 serum-free expression medium and transfected with the pTT3 vector containing the appropriate gene, as previously described [30]. Cells were centrifuged 96 h post-transfection and the culture medium was harvested, filtered by a 0.22 µm filter; the supernatants were concentrated twenty times using a 10 kDa cut-off Vivaflow 200 System (Vivasciences). All proteins were purified on a HisTrap Fast Flow Ni-affinity column (GE Healthcare). Proteins were further purified by ion exchange chromatography on an SP Sepharose column (GE Healthcare) followed by gel filtration chromatography on a Superdex 200 16/60 column (GE Healthcare) in 50 mM sodium phosphate buffer pH 6.8, 200 mM NaCl. Proteins were then concentrated using Macro- and Microsep concentrators (Millipore).

#### (ii) Escherichia coli Recombinant Protein Expression and Purification

The gene encoding FCR3-DBL3X (residues 1218–1577), cloned into a modified pET15b vector, was a kind gift from Dr. Matthew K. Higgins; expression was carried out as previously described [37]. The gene encoding 3D7-DBL5ε was PCR amplified and cloned into the pET15b vector between the NdeI/XhoI restriction sites, in frame with the N-terminal hexa-His tag, using the following oligonucleotide primers DBL5εF (5′-ACTGGCAG catatg TCC AAG ATG AAG GTG TGC GAC C-3′) and DBL5εR (5′-GCAG ctcgag TTA CAT GTC ATT GAA GCC GCA GGG G -3′). The gene encoding FCR3-DBL3X-4ε was PCR amplified and cloned into a modified pET21b vector using the following oligonucleotide primers FCR3-DBL3XF (5′-ACTGGCAG gctagc ATG AGC GAG ACC AGC TGC GAC C-3′) and FCR3-DBL4εR (5′-ACTGGCAG gcggccgc TCA ATG ATG ATG ATG ATG ATG CAT CTT GTT CAG CAC CTC GTT C -3′). Similarly, genes encoding 3D7-DBL2X and 3D7-DBL1X-2X were PCR amplified and cloned into a modified pET21b vector in frame with a C-terminal hexa-His tag using the following oligonucleotide primers DBL2XF (5′- GCAG gaattc CAG GAT TTC CTG CGC ATT CTG-3′), DBL2XR (5′-GGCAG gtcgac TTA ATG ATG ATG ATG ATG ATG ATT GGT AGG GAT TTT GCA CTG G -3′) and DBL1X-2XF (5′- GCAG gaattc TGT AAG ATC ACA GTG AAC CAC AGC GAT TCC GGC ACA AAT GAT CCT TG -3′) and DBL1X-2XR (5′-GGCAG gtcgac TTA ATG ATG ATG ATG ATG ATG ATT GGT AGG GAT TTT GCA CTG G -3′).

All proteins were expressed either in the Rosetta-Gami, Origami B or SHuffle strains of *E. coli* (Novagen) as soluble proteins at 20°C for 20 h after IPTG induction. Post-induction cells were centrifuged, resuspended in 20 mM Tris-HCl, 150 mM NaCl, pH 7.5 and lysed with an Emulsiflex homogeniser (Avestin). The proteins were purified using a metal affinity column (TALON, Clontech). All proteins were further purified using a heparin affinity column (GE Healthcare) and were eluted in 20 mM Tris-HCl pH 7.5, 1 M NaCl, followed by gel filtration (Sephedex 75 16/60, GE Healthcare) in 20 mM Tris-HCl 150 mM NaCl, pH 7.5.

### ELISA binding assays of recombinant proteins to various sulfated glycosaminoglycans

ELISA binding assays were performed as previously described [34]. Briefly, ELISA plates were coated overnight at 4°C with different sulfated glycosaminoglycans (GAG): 5 µg/mL for decorin (Sigma, D8428); 50 µg/mL for chondroitin sulfate A (CSA) (Sigma, C8529), chondroitin sulfate C (CSC) (Seikagaku, 400670) and heparan sulfate (HS) (Sigma, H7640) in PBS (Gibco, NaCl 155 mM pH 7.2), using 100 µL per well. BSA at 1% in PBS was used as background measurement. After coating, the wells were blocked with 150 µL of dilution buffer per well (PBS 1% BSA, 0.05% Tween20) for 1 h at 37°C. After removal of the blocking solution, each recombinant protein (3D7-DBL1X, 3D7-DBL2X, FCR3-DBL3X, 3D7-DBL5ε, 3D7-DBL1X-2X, FCR3-DBL3X-4ε, 3D7-DBL1X-CIDR, 3D7-DBL1X-3X, 3D7-DBL1X-4ε, 3D7-DBL1X-5ε and 3D7-DBL1X-6ε) at serial dilutions of 0.3125–20 µg/mL in the dilution buffer, was added per well and incubated for 1 h at 37°C with gentle shaking. After washing three times with PBST (PBS containing 0.05% Tween 20), 100 µL anti-His HRP conjugated antibody (diluted 1/2000 in dilution buffer) was added to each well and incubated for 1 h at 37°C. After washing three times with PBST, the interaction was quantified with TMB (3,3′,5,5′-tetramethylbenzidine) substrate (Biorad) using 100 µL per well for 20 min or until saturation was reached. Absorbance was measured at 655 nm.

### Adhesion inhibition assays of recombinant proteins to CSPG

Inhibition assays were performed using a protocol similar to that described above for ELISA, with decorin coated on the plate. Recombinant proteins (3D7-DBL1X-2X, 3D7-DBL1X-CIDR, 3D7-DBL1X-3X, 3D7-DBL1X-4ε, 3D7-DBL1X-5ε and 3D7-DBL1X-6ε) at a concentration of 1 µg/mL were premixed with increasing amounts of BSA, CSA, CSC, or HS (0.156–100 µg/mL) and incubated for 30 min at room temperature with gentle shaking before addition to the coated ELISA plate.

### Surface Plasmon Resonance

Interaction between the recombinant proteins and human placental CSPG was studied by surface plasmon resonance (SPR) using a Biacore® 2000 system (GE Healthcare) as previously described [34]. Human placental CSPG (MR4 Reagents Resource) was covalently coupled via primary amino groups of the protein moiety to the sensor chip (CM5 chip, GE Healthcare) surface using amine coupling kit (GE Healthcare) as described previously [38]. The amount of immobilized CSPG corresponded to 240 response units (RU). A separate flow channel on the same sensor chip without CSPG was used for control runs. For all SPR measurements, the recombinant domains were dialyzed against PBS buffer (Gibco), 0.005% P20 (GE Healthcare), and centrifuged immediately before the runs to minimize possible effects from nonspecific aggregation. The association was monitored by injecting different concentrations of the DBL analytes at 25°C with the flow rate of 20 µL/min for 300 s to achieve steady-state binding. Between each injection, surfaces were regenerated using 10 µL of 2 M NaCl followed by 10 µL of SDS 0.05%. All curves were corrected for nonspecific binding by subtraction of control curves obtained from injection of the corresponding protein through the blank flow channel. K_D_ was determined from the concentration dependence of steady-state SPR response (after corrections for nonspecific binding) using the Biacore BIAevaluation 3.1 software (Biacore AB).

### Animal immunization

Immunizations with 3D7-DBL1X-6ε VAR2CSA recombinant protein were performed at Proteogenix, France, according to animal immunization guidelines. Briefly, a New Zealand white rabbit was immunized with recombinant protein in TiterMax Gold Adjuvant (Sigma) intradermally with 50 µg of immunogen for first immunization and subcutaneously with 25 µg of immunogen in subsequent injections. IgG were purified from rabbit plasma using Hitrap protein G (GE Healthcare) according to manufacturer's instructions.

### IgG purification and depletion

The recombinant proteins (3D7-DBL1X, 3D7-DBL2X, FCR3-DBL3X, 3D7-DBL5ε, 3D7-DBL6ε, 3D7-DBL1X-2X, 3D7-DBL1X-CIDR, 3D7-DBL1X-3X, FCR3-DBL3X-4ε, 3D7-DBL1X-4ε, 3D7-DBL1X-5ε and 3D7-DBL1X-6ε) were coated on M-280 tosyl-activated Dynabeads (Invitrogen) according to manufacturer's instructions. Briefly, 66 µL of M-280 tosyl-activated Dynabeads were washed three times in 900 µL PBS, pH 7.4, and resuspended in the same buffer. Beads were then mixed with 20 µg of each recombinant protein containing 1.2 M ammonium sulfate and incubated for 12 h at 37°C with slow rocking. Following the incubation, the beads were washed five times with 1 mL PBS, incubated 1 h at room temperature with PBS, 0.5% BSA and washed 2 times in PBS and stored at 4°C in the same buffer.

For antibody depletion, 50 µL of purified rabbit anti 3D7-DBL1X-6ε IgG at 160 µg/mL diluted in PBS were incubated for 1 h at 37°C with the different protein-coated beads. Unbound antibodies were collected and beads were washed three times with 1 mL PBS prior to elution of bound antibodies using 50 µL 0.1 M glycine pH 2.5. Eluted fractions were subsequently neutralized with Tris-HCl 1 M pH 8.0.

Inhibition assays were performed using a protocol similar to that described above for ELISA using decorin-coated plates. Recombinant proteins at a concentration of 0.25 µg/mL were premixed with either the unbound antibody fractions or the eluted antibody fractions, and incubated for 30 min at room temperature with gentle shaking before addition to the coated ELISA plate.

## Results

### Recombinant DBL domains were successfully purified to high purity

Var2CSA is a 350 kDa protein possessing a large extracellular region, a transmembrane region and a short, conserved intracellular domain (Fig. 1A). While individual domains of var2CSA have been expressed and purified in various heterologous expression systems [29], [30], [33], [39], [40], it has been difficult to produce multiple PfEMP1 domains due to their large size and the presence of many cystine bridges. Recently, we reported the expression of the complete extracellular region of var2CSA using the human embryonic kidney cell (HEK293) heterologous expression system [34]. In the present study, we attempted to express in the HEK293 heterologous expression system various single and multiple domain recombinant proteins from the 3D7 var2CSA ortholog (3D7-DBL1X, 3D7-DBL1X-CIDR, 3D7-DBL1X-3X, 3D7-DBL1X-4ε, 3D7-DBL1X-5ε and 3D7-DBL1X-6ε). In addition, single (3D7-DBL2X, FCR3-DBL3X, 3D7-DBL5ε) and double (3D7-DBL1X-2X, FCR3-DBL3X-4ε) DBL domains were expressed as soluble proteins in *E. coli*. All these proteins were successfully expressed and purified to better than 95% homogeneity (Fig. 1B). Some domains, such as DBL4ε, could not be obtained due to poor expression yields. In SDS-PAGE under reducing conditions, all proteins migrated according to their expected molecular weights: 3D7-DBL1X (45 kDa), 3D7-DBL2X (53 kDa), FCR3-DBL3X (43 kDa), 3D7-DBL5ε (37 kDa), 3D7-DBL1X-2X (105 kDa), FCR3-DBL3X-4ε (86 kDa), 3D7-DBL1X-CIDR (134 kDa), 3D7-DBL1X-3X (176 kDa), 3D7-DBL1X-4ε (215 kDa), 3D7-DBL1X-5ε (259 kDa), and 3D7-DBL1X-6ε (300 kDa) (Fig. 1C), while under non-reducing conditions a shift in the migration of all proteins confirmed the presence of disulfide bridges (Fig. 1B). N-terminal sequencing and Western blots using anti-His antibodies confirmed that the proteins were not degraded (data not shown).

The yields after purification varied from 0.1 to 20 mg per litre of culture media: 3D7-DBL1X (1 mg/L), 3D7-DBL2X (5 mg/L), FCR3-DBL3X (20 mg/L), 3D7-DBL5ε (1 mg/L), 3D7-DBL1X-2X (1 mg/L), FCR3-DBL3X-4ε (3 mg/L), 3D7-DBL1X-CIDR (0.25 mg/L), 3D7-DBL1X-3X (0.25 mg/L), 3D7-DBL1X-4ε (0.15 mg/L), 3D7-DBL1X-5ε (0.15 mg/L), and 3D7-DBL1X-6ε (0.5 mg/L).

### Minimal CSA-binding region lies within 3D7-DBL1X-3X

**Figure 2:**
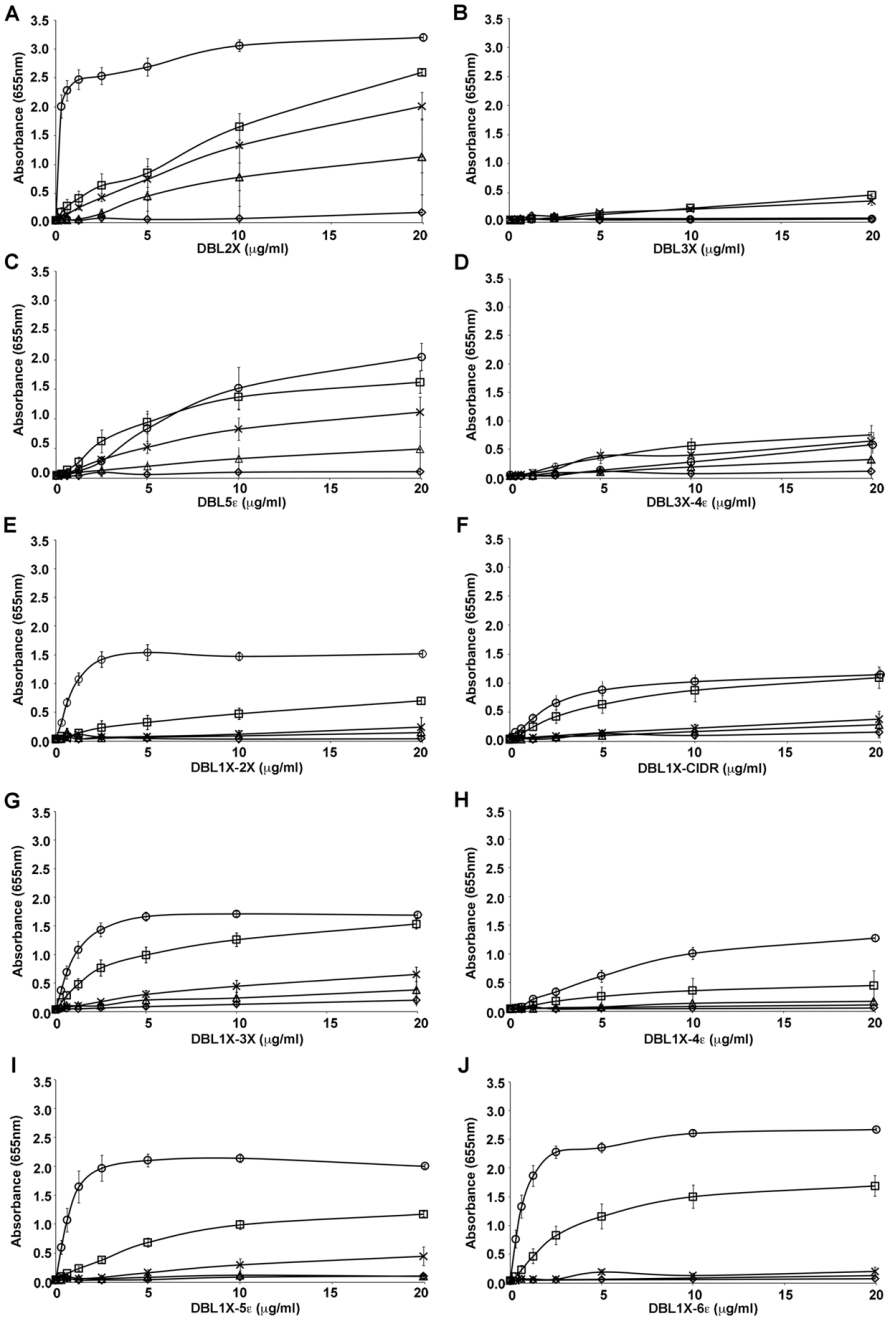
Binding of recombinant DBL domains from 3D7 and FCR3-DBL1X-6ε to different glycosaminoglycans. ELISA-based direct binding assay was performed to identify the specificity of (A) 3D7-DBL2X, (B) FCR3-DBL3X, (C) 3D7-DBL5ε, (D) FCR3-DBL3X-4ε, (E) 3D7-DBL1X-2X, (F) 3D7-DBL1X-CIDR, (G) 3D7-DBL1X-3X, (H) 3D7-DBL1X-4ε, (I) 3D7-DBL1X-5ε and (J) 3D7-DBL1X-6ε to different sulfated glycosaminoglycans. Increasing concentrations of recombinant proteins at serial dilutions of 0.31–20 µg/mL were added to wells previously coated with BSA (▵) or different glycosaminoglycans: decorin (○), CSA (□), CSC (⋄), HS (×).

The adhesion properties of the different recombinant proteins were tested by direct ELISA using plates coated with different sulfated glycosaminoglycans (GAG), including chondroitin sulfate A (CSA), chondroitin sulfate C (CSC), decorin (a proteoglycan containing CSA), and heparan sulfate (HS) (Fig. 2A–J). Among the single DBL domains tested 3D7-DBL1X did not bind to any of the sulfated GAGs (data not shown) and 3D7-DBL3X bound weakly to HS and CSA at higher concentrations (Fig. 2B), like DBL6ε as previously reported [34]. Interestingly, 3D7-DBL2X and 3D7-DBL5ε bound to decorin and CSA in a dose-dependent manner but these proteins also bound to BSA and heparan sulfate at higher concentrations (Fig. 2A and C), indicating some non-specific interactions. For 3D7-DBL2X, a much stronger signal was observed in binding to decorin in comparison to CSA, reaching saturation at very low concentrations. Binding properties of the double domain recombinant proteins were also tested. While DBL1X-2X bound with high affinity to decorin, the apparent affinity to CSA was lower (Fig. 2E). DBL3X-4ε bound to the different GAGs in a weak, non-specific manner (Fig. 2D). Similarly, recombinant proteins possessing more than two domains (3D7-DBL1X-CIDR, 3D7-DBL1X-3X, 3D7-DBL1X-4ε and 3D7-DBL1X-5ε) were tested for their binding behavior with CSA and were compared with the full-length 3D7-DBL1X-6ε protein. All multiple domain proteins bound to decorin and CSA in a dose-dependent manner with various degrees of affinity, except for DBL1X-4ε which did not bind to CSA (Fig. 2F–J). DBL1X-3X and DBL1X-5ε exhibited an affinity and specificity quite similar to the full-length 3D7-DBL1X-6ε protein while 3D7-DBL1X-CIDR had lower affinity to CSA and decorin.

Our results indicate that the high affinity CSA-binding site lies within the DBL1X-3X recombinant protein.

**Figure 3:**
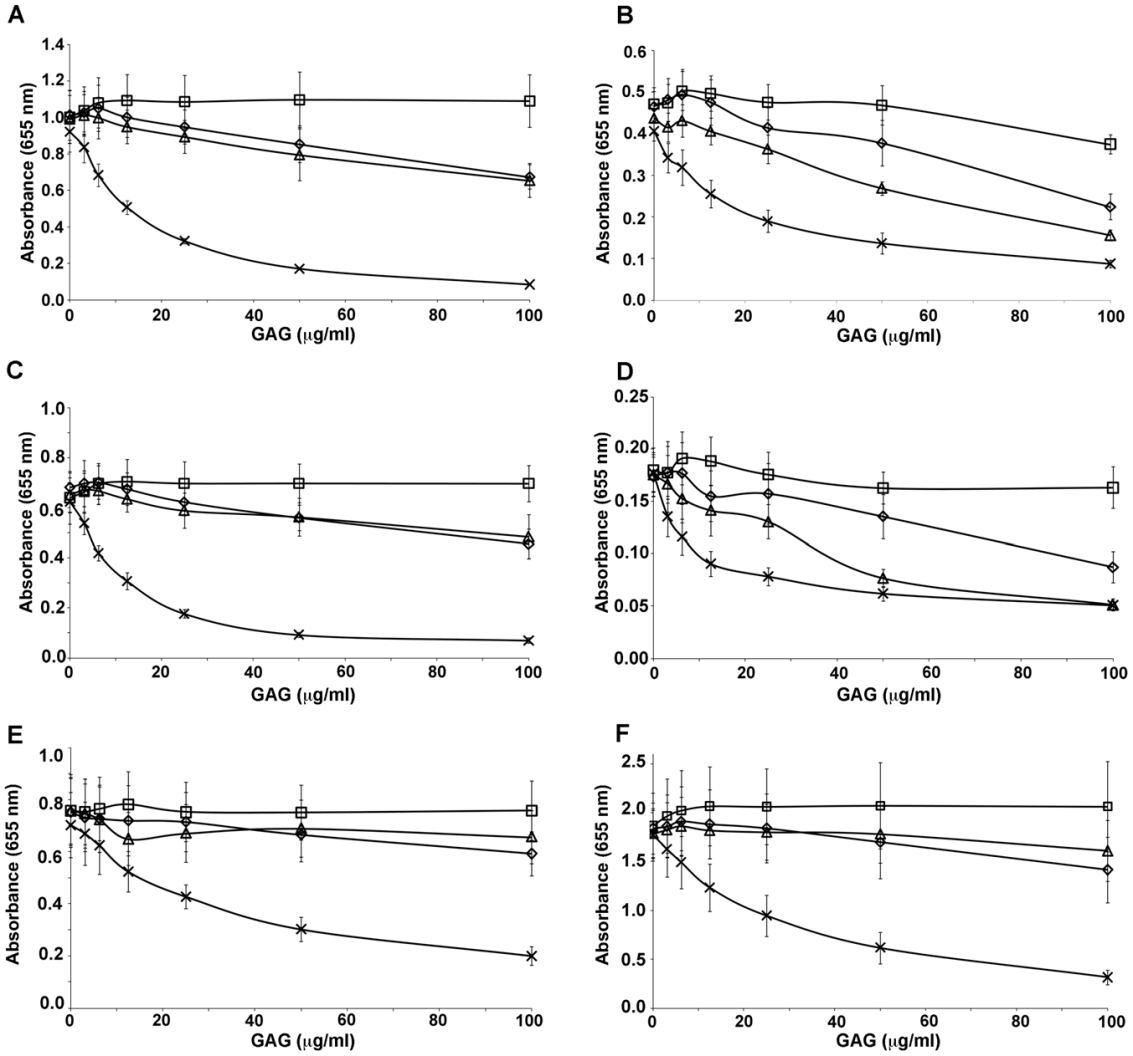
Competitive inhibition of recombinant DBL domains binding to decorin using various glycosaminoglycans. Recombinant proteins (A) 3D7-DBL1X-2X, (B) 3D7-DBL1X-CIDR, (C) 3D7-DBL1X-3X, (D) 3D7-DBL1X-4ε, (E) 3D7-DBL1X-5ε and (E) 3D7-DBL1X-6ε at 1 µg/mL were premixed with increasing amounts of BSA (□) or glycosaminoglycans, 0.156–100 µg/mL of CSA (×), CSC (⋄) or HS (▵) and incubated in plates previously coated with decorin.

To further assess the binding specificity of the multidomain recombinant proteins, we tested whether different sulfated GAGs could compete with decorin in binding to these proteins. Decorin-coated plates were incubated with a constant concentration of recombinant protein premixed with increasing concentrations of CSA, CSC, or HS. Binding of all the recombinant proteins (3D7-DBL1X-2X, 3D7-DBL1X-CIDR, 3D7-DBL1X-3X, 3D7-DBL1X-4ε 3D7-DBL1X-5ε and 3D7-DBL1X-6ε) to decorin was inhibited by CSA in a dose-dependent manner. Significant inhibition with the other sulfated GAGs, was also observed at higher concentrations for 3D7-DBL1X-CIDR, and 3D7-DBL1X-4ε (Fig. 3B and D). However, the binding of 3D7-DBL1X-2X and 3D7-DBL1X-3X (Fig. 3A and C) to decorin was specifically and efficiently inhibited by CSA and most closely mimicked the binding inhibition of DBL1X-5ε and DBL1X-6ε (Fig. 3E and F).

Taken together, our results indicate that the minimal region within var2CSA for specific and high affinity binding to CSA lies within DBL1X-3X and that the binding site is mainly centred on the DBL2X domain. Although 3D7-DBL2X binds with high affinity to decorin and CSA, it shows poor specificity as it also binds to other GAGs at higher concentrations (Fig. 2A). Thus, with our observations that DBL1X does not bind to any GAG (data not shown) and that DBL3X binds weakly to HS and CSA (Fig. 2B), we propose that the main CSA-binding residues are likely to be present on DBL2X and that the surrounding domains, DBL1X, CIDR and DBL3X, contribute to the affinity and specificity of this interaction by a higher-order organisation of the multidomain protein.

### N-terminal multidomain constructs bind placental CSPG with high affinity

**Figure 4:**
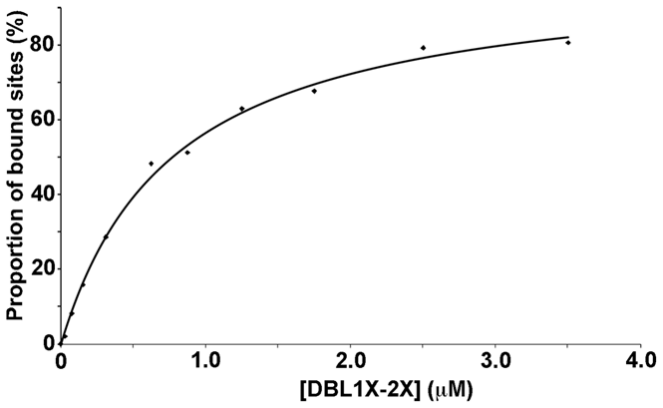
Determination of affinity constants for binding of DBL1X-2X to human placental CSPG. Surface plasmon resonance binding experiments were performed with placental CSPG immobilized on a sensor chip and soluble proteins as analyte. The variation in the steady-state SPR signal as a function of protein concentrations (from 0.031 µM to 3.5 µM) was used to calculate the dissociation constant K_D_ (Table 1).

The binding of the recombinant proteins 3D7-DBL2X, 3D7-DBL1X-2X, 3D7-DBL1X-CIDR, 3D7-DBL1X-3X, 3D7-DBL1X-5ε and 3D7-DBL1X-6ε to placental CSPG was examined more quantitatively by real-time SPR. Data from the kinetic association and dissociation curves did not fit a simple 1∶1 binding model, thus precluding the use of kinetic constants k_on_ and k_off_ to estimate the binding constant K_D_. We therefore performed concentration-dependent, steady-state SPR experiments to determine the constants K_D_, which were calculated from the variation of the steady-state binding response with protein concentration, as shown for 3D7-DBL1X-2X (Fig. 4).

Results are summarized in Table 1, which also includes previously published constants for other var2CSA-derived recombinant proteins for comparison. Not all domains could be tested since the expression yields of some were not sufficient for SPR experiments.

**Table 1:** K_D_ for var2CSA recombinant proteins binding to placental CSPG.

*Protein*	*K_D_ (µM)*
3D7-DBL2X	5.3
FCR3-DBL3X	344*
CYK39-DBL5ε	150#
3D7-DBL6ε	92*
3D7-DBL1X-2X	0.77
3D7-DBL1X-CIDR	1.58
3D7-DBL1X-3X	0.58
3D7-DBL1X-5ε	0.115
3D7-DBL1X-6ε	0.127

K_D_ values were determined from the concentration dependence of steady-state SPR response using the Biacore BIAEVALUATION 3.1 software.

*: from reference [34].

# : from reference [41].

The calculated affinity for 3D7-DBL1X-5ε was similar to that of 3D7-DBL1X-6ε indicating that DBL6ε has little or no role in the adhesion process. The affinities of 3D7-DBL1X-2X and 3D7-DBL1X-3X for human placental CSPG are ∼5 times lower than that of 3D7-DBL1X-6ε, but ∼10 times higher than that of 3D7-DBL2X and ∼150 times higher than that of the single DBL domains FCR3-DBL3X and FCR3-DBL6ε. K_D_ for the binding of DBL5ε, derived from the placental strain CYK39, for placental CSPG was reported as 140 µM [41], similar to that of the single 3D7-DBL3X and 3D7-DBL6ε domains. Thus, 3D7-DBL1X-2X is the shortest var2CSA recombinant protein with an affinity for human placental CSPG that is the most comparable with that of 3D7-DBL1X-6ε.

### DBL3X and DBL5ε possess epitopes for inhibitory antibodies

**Figure 5:**
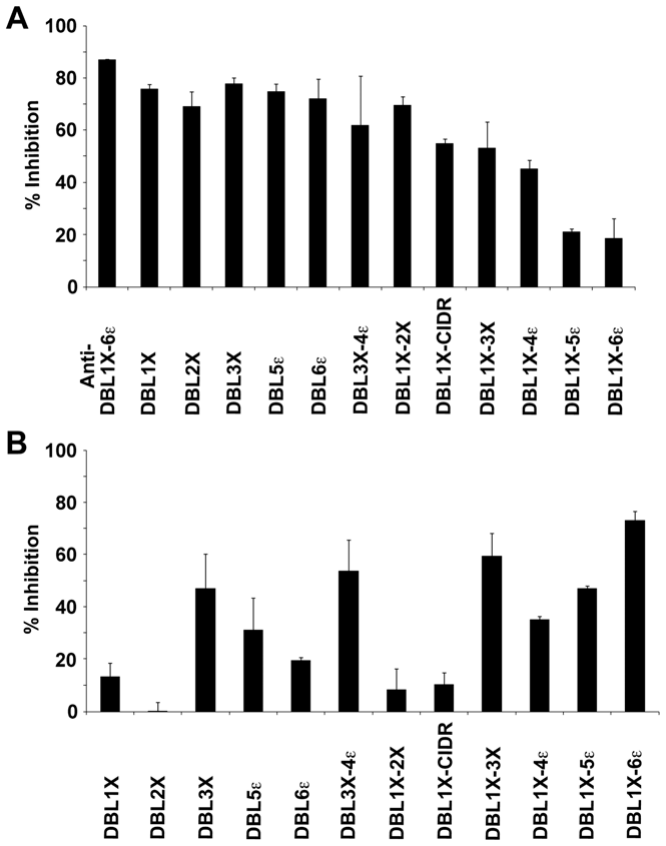
Depletion of purified adhesion-inhibitory rabbit IgGs (anti 3D7-DBL1X-6ε) by various recombinant proteins. Immunization of rabbit with 3D7-DBL1X-6ε induces adhesion-blocking antibodies. Purified IgG against 3D7-DBL1X-6ε (Anti-DBL1X-6ε was depleted on 3D7-DBL1X, 3D7-DBL2X, FCR3-DBL3X, 3D7-DBL5ε, 3D7-DBL6ε, 3D7-DBL1X-2X, 3D7-DBL1X-CIDR, FCR3-DBL3X-4ε, 3D7-DBL1X-3X, 3D7-DBL1X-4ε, 3D7-DBL1X-5ε and 3D7-DBL1X-6ε immobilized on tosylactivated beads. Unbound (Fig. 5A) and bound (Fig. 5B) fractions were tested for inhibition of binding of 3D7-DBL1X-6ε to decorin. Data are express as % of inhibition compare to control.

Recently, we showed that immunization of rabbits with full-length var2CSA generated inhibitory antibodies capable of blocking var2CSA and PEs adhesion to CSA [42]. In order to determine which domains of var2CSA are preferentially targeted by these inhibitory antibodies, we used our var2CSA recombinant proteins immobilized on beads to deplete rabbit IgG; the depleted and protein-purified antibodies were then tested for their capacity to inhibit the interaction of the full-length protein with decorin. As expected, immobilized DBL1X-6ε was able to deplete the inhibitory activity of the var2CSA-purified IgG fraction very efficiently, as inhibition dropped from 85% to less than 20% (Fig. 5A). Furthermore, antibodies eluted from the beads were able to inhibit the interaction between DBL1X-6ε and decorin by 72%, almost completely recapitulating the initial level of inhibition. Among the single and multidomain var2CSA proteins, only DBL1X-CIDR, DBL1X-3X, DBL1X-4ε and DBL1X-5ε were able to significantly deplete an important fraction of inhibitory antibodies (Fig. 5A).

No significant inhibitory activity was observed in the fractions eluted from beads carrying DBL1X, DBL2X, DBL6ε, DBL1X-2X or DBL1X-CIDR (Fig. 5B). However, antibody fractions eluted from the single domains DBL3X and DBL5ε, as well as those eluted from the multidomains DBL3X-4ε, DBL1X-3X, DBL1X-4ε and DBL1X-5ε, had significant inhibitory activity ranging from 35% for DBL1X-4ε up to 59% for DBL1X-3X (Fig. 5B). Of note, although we were not able to express DBL4ε domain alone to assess its contribution, DBL3X-4ε was more effective than DBL3X and almost as effective as DBL1X-3X in retaining inhibitory antibodies, suggesting that DBL4ε could also be a target of inhibitory antibodies, as previously reported [32]. Taken together, these results indicate that although the DBL1X-DBL2X region is central in forming the high affinity CSA-binding site, no inhibitory antibodies were generated against these domains, while most of the inhibitory antibodies targeted DBL3X and, to some extent, DBL5ε.

## Discussion

Pregnancy-associated malaria is the consequence of *Plasmodium falciparum* PE sequestration in the intervillous space of placenta [1] through the CSA moiety of placental CSPG [3]. This interaction is mediated by the var2CSA PfEMP1 variant which is structurally unique among all *var* genes in the parasite genome and has been reported to contain multiple distinct CSA-binding domains (DBL2X, DBL3X, DBL5ε and DBL6ε), suggesting that multivalency may be important for placental sequestration [14], [26], [27]. Since var2CSA is central in this sequestration process, it is the main target for the development of effective drug and vaccine strategies. However, due to its high molecular weight and cysteine content, high-level expression of the full-length var2CSA protein is difficult to obtain, limiting most of the studies that aim to assess the breadth of antibody reactivity and inhibitory activity generated by single domain-based immunogens. Although single-domain immunization has, in many cases, generated good reactivity against various placental parasites, few have induced anti-adhesive antibodies [30], [31], [32], the best recombinant proteins being an IT4-DBL4ε var2CSA produced in baculovirus/insect cell system [32] and a refolded *E. coli* IT4-DBL5ε [43]. However, adhesion inhibitory activities are not consistent between various DBL4ε and DBL5ε antigen preparations [31], [44]. In general, therefore, this empirical approach of attempting to generate highly cross-reactive and inhibitory antibodies against a large panel of placental parasites appears to be unsuccessful and a more rational approach needs to be employed.

Recent work suggests that a high-affinity, CSA-specific binding site is formed by the higher-order domain organization of the var2CSA extracellular region. Indeed, unlike individual DBL domains, the full-length var2CSA extracellular region binds with high affinity and specificity to CSA [34], [35]. Structural characterization by analytical ultracentrifugation and small angle X-ray scattering has revealed a compact organization of the full-length var2CSA extracellular region (most likely governed by specific inter-domain interactions) rather than an extended structure [34]. Furthermore, animals immunized with DBL1X-6ε generate antibodies that are broadly strain-transcendent but do not cross-inhibit different placental-type parasite isolates [42]. Although it is now possible to express the full-length var2CSA, difficulties due to polymorphism and to scaling up of the production make it of limited utility as a vaccine candidate. Hence there is a need to identify the minimal binding region of var2CSA that retains the high affinity and specificity of the full-length protein, but also generates a broad inhibitory antibody response.

In this study, we have expressed various truncated var2CSA recombinant proteins in order to map the minimal binding region within the full-length protein that can mimic its high affinity and specificity. As reported previously, none of the single domains tested in this study bind specifically to CSA. Among these single domains, DBL2X had a strong interaction with decorin but it also bound other GAGs in a non-specific manner. Although the affinity of DBL1X-DBL2X for human placental CSPG was about five fold lower than for the full-length protein, it remained in the nanomolar range and displayed a specificity similar to the full-length protein. Our results indicate that DBL2X forms the central core of the binding site and that the high affinity and specificity of the var2CSA recombinant proteins are only achieved when DBL1 at least, is added as flanking region to the construct. Furthermore, the addition of the CIDR-DBL3X domain tends to increase even further the affinity and specificity of DBL1X-3X. This increase could be due to a stabilizing effect of DBL3X on the recombinant protein and the binding site. In addition, this domain could also contain residues directly involved in the interaction with CSA. However, DBL1X-4ε (which includes DBL1X-3X) did not bind to CSA, suggesting that the added DBL4ε domain, in the absence of the remaining C-terminal domains, may not adopt the correct higher-order configuration to form the native CSA-binding site. Our data also support a role for DBL5ε in stabilizing the CSA binding site, which could indicate that this domain is in close proximity to the binding site. Taken together, our results suggest that the high affinity CSA binding site lies within DBL1X-3X and that interactions between these domains and the domains outside of it are important for its stability.

The depletion/elution experiments indicate that DBL3X is one of the main targets of inhibitory antibodies, as only constructs carrying this domain were able to significantly deplete an important fraction of these antibodies. More importantly, purified antibodies eluted from these constructs, including the DBL3X single domain, possess significant adhesion-inhibitory properties ranging from 45% to 60%. Surprisingly, DBL1X-4ε and DBL1X-5ε were less efficient in retaining inhibitory antibodies than DBL1X-3X and even DBL3X alone, but this may reflect subtle effects of inter-domain interactions on the higher-order structure of the various multi-domain proteins, which, in the absence of structural data, we are unable to assess. Although the CSA binding site lies within the DBL1X-DBL2X region, no significant inhibitory activity was observed using eluted fractions from DBL1X, DBL2X and DBL1X-2X. This might indicate that, in the context of the full-length protein, the central binding core is not sufficiently exposed to induce an antibody response against important residues involved in CSA adhesion and that the inhibitory antibodies may be acting by steric hindrance. This hypothesis is supported by our observations that the single domain DBL5ε also retained inhibitory antibodies and that DBL3X-4ε was more effective than DBL3X, and almost as effective as DBL1X-3X, in retaining inhibitory antibodies, suggesting that DBL4ε is also a target of inhibitory antibodies, as previously reported [32]. In line with this, human monoclonal antibodies isolated from affinity-matured memory B cells of *P. falciparum*-exposed women were shown to recognize the DBL3X and DBL5ε domains and also inhibit var2CSA binding to CSA [45]. Similarly, it was recently reported that animals immunized with DBL5ε could induce adhesion blocking antibodies [43].

In conclusion, our study clearly indicates that the minimal CSA-binding region of var2CSA lies within DBL1X-3X and that inhibitory antibodies mainly target DBL3X and, to a lesser extent, DBL5ε. Studies of the immunogenic properties of DBL1X-3X and DBL3X-DBL5ε are in progress to provide further information on the role of these domains in CSA interaction and in generating inhibitory antibodies. Structural studies on the DBL1X-3X domain organization would provide additional insight into critical residues involved in CSA adhesion, permitting rational vaccine development strategies. This study is a step further in the development of effective drugs and vaccine strategies aiming to fight PAM.
